# Using the critical path method to rollout and optimise new PMTCT guidelines to eliminate mother-to-child transmission of HIV in Zimbabwe: a descriptive analysis 

**DOI:** 10.1186/s12913-020-05900-4

**Published:** 2020-11-13

**Authors:** Reuben Musarandega, Joanna Robinson, Priti Dave Sen, Anna Hakobyan, Angela Mushavi, Agnes Mahomva, Godfrey Woelk

**Affiliations:** 1 Elizabeth Glaser Paediatric AIDS Foundation (EGPAF), Block 5, Arundel Office Park, Mt Pleasant, Harare, Zimbabwe; 2 Children’s Investment Fund Foundation (CIFF), London, UK; 3 Ministry of Health and Child Care (MoHCC), Harare, Zimbabwe; 4 Elizabeth Glaser Paediatric AIDS Foundation, Washington DC, USA

**Keywords:** Critical path method, PMTCT, Elimination, Paediatric HIV, Vertical transmission, Quality improvement

## Abstract

**Background:**

Achievement of the elimination target for mother-to-child transmission (MTCT) of HIV in selected countries has increased hope to end the HIV epidemic in children across the world. However, MTCT rates remain well above the 5% elimination target in most sub-Saharan Africa countries. These countries require innovative strategies to scale-up their interventions to end paediatric HIV. We describe how the Elizabeth Glaser Paediatric AIDS Foundation (EGPAF) consortium and the Children’s Investment Fund Foundation (CIFF) used the critical path method to facilitate rapid expansion and optimization of 2010 and 2013 WHO PMTCT guidelines to reduce Zimbabwe’s MTCT rate from 22% in 2010 to 6.4% in 2015.

**Methods:**

We analysed activities implemented and PMTCT programme data for the period before and during the EGPAF-CIFF project. The critical path method involved a cycle of collecting and analysing quarterly PMTCT indicator data and planning and implementing targeted activities to improve the PMTCT indicators. We performed a graphical trend analysis of data that measured availability of PMTCT services. Using Pearson’s Chi2 test, we compared results of PMTCT uptake indicators at the start and end of the EGPAF-CIFF project and used regression discontinuity analysis to assess effectiveness of activities implemented to improve the PMTCT service uptake indicators.

**Results:**

Zimbabwe rolled out WHO 2010 and 2013 PMTCT guidelines in less than 1 year during the EGPAF-CIFF project, yet it took more than 4 years to roll-out previous guidelines. All PMTCT indicators increased significantly (*p* < 0.001) comparing the five-year periods before and during the EGPAF-CIFF project. Critical path activities implemented increased five of the seven PMTCT uptake indicators.

**Conclusion:**

Zimbabwe rapidly rolled-out and optimised new WHO PMTCT guidelines and drastically reduced its MTCT rate using the critical path method. We recommend wider use of the critical path method in public health programmes.

## Background

Achievements in prevention of mother-to-child transmission (PMTCT) of HIV in some countries have provided hope to ending the HIV epidemic in children across the world. Countries like Belarus, Cuba, Thailand, Malaysia and Armenia have reached the elimination thresholds for mother-to-child transmission (MTCT) of HIV and syphilis [1]. However, HIV MTCT rates are still well above the Joint United National Programme on AIDS (UNAIDS) elimination target of less than 5% in most sub-Saharan Africa countries [2]. In this region, innovative strategies are required to scale up and optimise PMTCT services to achieve the elimination of mother-to-child transmission of HIV [2–5].

PMTCT includes a cascade of services given to HIV-positive women and their HIV-exposed infants to prevent the transmission of HIV from the mother to the infant in-utero, during delivery and during breastfeeding. PMTCT services include HIV testing and counseling of pregnant women during antenatal care (ANC), labour and breastfeeding and providing antiretroviral (ARV) drugs to the HIV-positive mother and HIV-exposed infant [1–3, 6–8].

Zimbabwe started piloting PMTCT using the World Health Organization (WHO) 2001 PMTCT guidelines. The guidelines recommended HIV-positive pregnant women to receive Zidovudine (AZT) for 4 weeks or a single dose of Nevirapine (sd-NVP) at the onset of labour. The country adopted the sd-NVP option at that time. Zimbabwe skipped WHO 2004 guidelines and adopted 2006 guidelines. These recommended pregnant women to receive AZT daily from 28 weeks’ gestation until delivery plus sd-NVP in labour and 7 days AZT + 3TC tail after delivery or to initiate ART on CD4 threshold of 200 cells/μls of blood. Zimbabwe next adopted 2010 (“Option A”) and 2013 (“Option B+”) guidelines. “Option A” recommended HIV-positive pregnant women with WHO disease stage 3 & 4 or CD4 count ≤350 cell/μls to initiate ART from diagnosis while ART-ineligible women received AZT from 14 weeks’ gestational age and sd-NVP in labour. The 2013 guidelines recommended initiation on ART of all HIV-positive pregnant women in endemic areas for the duration of the breastfeeding period (“Option B”) or life-long ART (“Option B+”), irrespective of CD4 count or disease staging. HIV-exposed infants (HEI) received daily NVP syrup for 6 weeks in 2001, 2006 and 2013 guidelines, and for the breastfeeding period in 2010 guidelines. Before 2011, EGPAF supported a gradually increasing number of facilities from one in 2001, using strategies of employing few national level technical staff who trained facility nurses to provide PMTCT services and conducted regular support visits to the sites, as explained in Perez and others [9]. By 2010, EGPAF supported 815 PMTCT sites in 38 districts. Government provided PMTCT services in most of the remaining facilities, without partner support. Few other partners provided support to a small number of health facilities, by training nurses and laboratory staff on PMTCT and EID. Some partners procured the PMTCT ARVs [10, 11].

The critical path (CP) concept, used in this project, originated in the 1950s in large defense projects [12]. The CP is a sequence of dependent steps of a project, which takes minimum time to complete. The critical path method (CPM) is a project management approach, which applies to complex projects that have multiple steps with a defined order [12]. The CPM links resources to tasks and optimises time taken to complete project activities [12]. Previously, the CPM was largely used in the construction industry. In one of the few cases of its use in health, Mount Clemens General Hospital in Michigan, USA, piloted the use of the CPM in health service total quality management, an approach that seeks to improve processes, products, services, and the culture of an organization to achieve customer satisfaction [13]. The United States Department of Health and Human Services and Agency for Healthcare Research and Quality also used the CPM as a tool for assessing workflow, i.e. assessing how health institutions organized health services, in order to improve the quality of health services [14]. Use of the CPM in health has since expanded to include coordinating implementation of programmes, containing programme costs, and improving clinical care processes, among other uses [15–18].

**Fig. 1:**
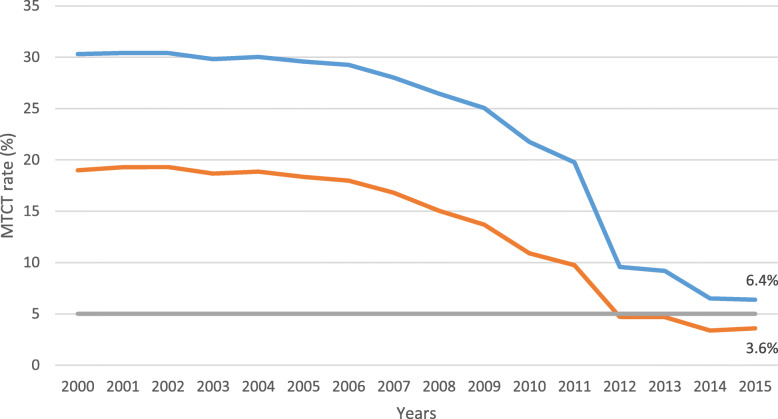
Zimbabwe MTCT rates, 2000–2015 [Source: MoHCC, HIV estimates report, 2016].  Final MTCT.  MTCT at 6 weeks.  Target < 5%

We describe how the Elizabeth Glaser Paediatric AIDS Foundation (EGPAF) and its consortium members (Organization for Public Health Interventions and Development, J. F Kapnek Trust and University of Zimbabwe – Zimbabwe AIDS Prevention Project) and the Children’s Investment Fund Foundation (CIFF) used the CPM to rapidly roll-out and optimise 2010 and 2013 WHO PMTCT guidelines in Zimbabwe, reducing the country’s MTCT rate to within the UNAIDS target of less than 5%. A population-based survey estimated Zimbabwe’s MTCT rate at 18 months after birth at 6.7% in 2014 and the Spectrum model estimated the 18-months MTCT at 6.4% in 2015 (Fig. 1) [19, 20]. The aim of this paper is to demonstrate the usefulness of the CPM in public health programmes. The paper contributes to the body of knowledge on the application of the CPM in health settings.

## Methods

### Study design and setting

In this retrospective, implementation science study, we describe the project activities implemented in applying the CPM and analysed Zimbabwe’s PMTCT service availability and uptake data for the period before (2006–2010) and during (2011–2015) the EGPAF-CIFF project. The project supported nearly all 1560 MNCH facilities in Zimbabwe to provide PMTCT services using current WHO guidelines.

### Project activities implemented

The EGPAF-CIFF consortium implemented several broad, strategic and specific critical path activities (CPAs) at national, district and facility levels to enhance the PMTCT programme (Table 1). CPAs were part of the CPM, and were adopted after analysing the data for each indicator in quarterly and annual programme reviews and were aimed to improve specific PMTCT indicators. Previous strategies for PMTCT programme support lacked this intense, regular programme performance analysis and implementation of targeted activities to improve programme indicators. The CPM guided resource allocation in activity budgets, where the project prioritized and funded activities linked to PMTCT indicators that needed to be improved in order to achieve the overall programme goal.

**Table 1:** Interventions implemented from 2011 to 2015 to improve critical path indicators and time of introducing them

Critical Path Indicator	Period when intervention was introduced		Intervention implemented
	Year	Quarter	
ANC Bookings	2011	Q2	Incorporated PMTCT messaging in VHW training materials, trained and engaged VHWs in community mobilization for PMTCT and conducted mass-media campaigns to promote early ANC bookings.
HIV Testing	2011	Q2	Trained more nurses to offer rapid HIV testing to pregnant women; tracked availability of test kits at site level and helped to redistribute as necessary to avoid stock outs.
Maternal AZT Prophylaxis	2011	Q2	Introduced integrated PMTCT curriculum which trained MNCH nurses to dispense AZT to HIV-positive pregnant women according to 2010 WHO guidelines.
CD4 Testing	2011	Q4	Procured 154 POC CD4 testing machines and distributed to MNCH clinics and EDTA tubes for remote clinics to collect and send blood specimens for CD4 testing at sites with CD4 testing machines.
Mothers’ ART	2011	Q4	Introduced ART in MNCH and trained MNCH nurses to initiate eligible HIV-positive pregnant women on ART rather than referring them to existing ART clinics for initiation by doctors.
Infant ARVs	2011	Q2	Changed HIV-exposed infants’ ARV prophylaxis from seven days daily AZT dose to daily NVP dose until 7 days after breastfeeding.
EID	2011	Q2	Incorporated EID training into PMTCT training materials and trained nurses in all MNCH facilities to offer EID services.
All indicators	2011	Q2	Quarterly data analysis, review of site level performance, implementing PDSA cycles to improve the indicators

*PMTCT* prevention of mother-to-child transmission, *VHW* Village health worker, *ANC* Antenatal care, *MNCH* Maternal, neonatal and child health, *AZT Zidovudine, POC* Point of care*, EDTA* Ethylenedia-minetetraacetic acid*, ART* Antiretroviral therapy, *NVP* Nevirapine*, EID* Early infant diagnosis*, DBS* Dried blood spot

At national level, the consortium seconded to the Ministry of Health and Child Care (MoHCC) a senior Paediatrician who coordinated the national PMTCT and Paediatric ART programme. The consortium assisted Zimbabwe to develop new PMTCT strategic plans, adapt WHO 2010 and 2013 guidelines into national PMTCT guidelines, develop PMTCT training manuals and standard operating procedures, update patient registers and reporting forms. Among interventions planned to improve different PMTCT indicators, the consortium helped the MoHCC to incorporate PMTCT content in village health workers (VHWs) training manuals [21]. The consortium procured and distributed point-of-care (POC) CD4 testing machines and Ethylenediaminetetraacetic acid (EDTA) tubes to MNCH facilities without laboratories to perform CD4 testing on-site or collect and send CD4 testing blood specimens to hospital laboratories [22]. The consortium funded a courier service, which expanded and optimised EID services countrywide and initiated a clinical mentorship programme to increase MNCH nurses’ confidence to initiate pregnant women and under-five children on ART [23]. The consortium initiated a quality improvement programme, which trained MNCH nurses to use plan-do-study-analyse (PDSA) cycles to improve PMTCT service uptake and quality in their facilities [24].

At district level, the consortium recruited, trained and deployed 37 senior nurses to the MoHCC district offices as PMTCT district focal persons (DFPs) [25]. It trained around 4500 ANC nurses in PMTCT and paediatric ART guidelines countrywide. The DFPs assisted District Nursing Officers (DNOs) to coordinate, implement and monitor PMTCT services in their districts. DFPs facilitated the clinical mentorship programme which involved one-week didactic training of nurses in ART management for pregnant and breastfeeding women and paediatric patients and two-week attachment at ANC facilities implementing nurse-led ART initiation. Medical doctors at centre-of-excellence hospitals conducted the trainings. DFPs and MoHCC’s community nurses trained around 3000 VHWs countrywide, who included previous PMTCT clients [21]. The VHWs used all forms of community gatherings to mobilize communities for PMTCT and encourage pregnant women to visit MNCH facilities for ANC and PMTCT services. DFPs visited each supported facility at least thrice per quarter to supervise and support PMTCT services.

At facility level, DFPs coached, mentored and assisted nurses to implement new PMTCT guidelines. DFPs assisted facility nurses to forecast, order, and redistribute PMTCT and paediatric ART medicines and dried blood spot (DBS) kits for EID to avert stock-outs and expiries in health facilities. They assisted nurses to analyse PMTCT data and use the data to develop PDSA cycles to improve the uptake and quality of PMTCT services in their facilities. DFPs assisted facility nurses to monitor retention of PMTCT clients in care. They helped facility nurses to compile lists of defaulting HIV-positive pregnant and breastfeeding women and HIV-exposed infants due for HIV early infant diagnosis (EID) and give to VHWs to track and bring back to care [25].

### Use of the critical path method in the project

EGPAF and CIFF developed the project’s critical path – a set of seven PMTCT uptake indicators also named critical path indicators (CPIs), which were number and percentage of women or infants (out of those eligible) receiving different PMTCT services. Seven programme management indicators (PMIs), which monitored the availability of PMTCT services at each health facility, complemented the CPIs. A logical framework linked the CPAs, PMIs, CPIs and project goal (Table 2). The project goal was to reduce Zimbabwe’s MTCT rate from as estimated 30% in 2010 to less than 12% by 2015. The baseline was based on UNAIDS’ Spectrum software MTCT rate estimates of 2009. Spectrum is a population projection software, which UNAIDS uses to generate estimates for different HIV indicators in different countries [26]. CIFF consultants developed annual targets for each CPI using an unpublished mathematical model, which predicted incremental uptake percentages to be attained year-on-year in each CPI to achieve an MTCT rate below 12% [27].

**Table 2:** Critical path logical framework

Critical path impact goal	To reduce the MTCT rate from an estimated 30% in 2010 to less than 12 in 2015
	↑
Critical path indicators (CPIs)	➢ Number and % women booked for first ANC visit (ANC booking is attending at least one ANC visit and is counted at the 1st ANC visit) ➢ Number and % women tested for HIV in ANC ➢ Number and % HIV-positive women started on AZT prophylaxis in ANC ➢ Number and % pregnant women eligible for ART by CD4 count in ANC ➢ Number and % eligible pregnant women initiated on ART in ANC ➢ Number and % HIV-exposed infants started on Nevirapine prophylaxis ➢ Number and % infants (< 2 months age) tested for HIV using DNA PCR
	↑
Programme management indicators (PMIs)	➢ Number of sites offering comprehensive PMTCT services (HIV testing and ARVs for PMTCT) ➢ Number of sites offering ART services on-site (in MNCH or ART clinic) ➢ Number of sites offering CD4 testing on-site (using point-of-care machine or on-site laboratory CD4 testing machine) ➢ Number of sites offering EID services on-site (collecting dried blood spot and sending to a central laboratory for testing) ➢ Number of health facility nurses trained on PMTCT, ART, EID etc. ➢ Number of sites with an interruption in HIV testing for at least one day (due to absence of trained rapid HIV testers or stock-out of HIV test kits) ➢ Number of sites with a stock-out of maternal and infant ARVs ➢ Number of sites visited for supportive supervision
	↑
Critical path activities	➢ Supporting programme coordination, adaptation of global PMTCT guidelines, developing training materials, technical working groups etc. ➢ Supporting rapid roll-out of new PMTCT guidelines in health facilities ➢ Training and mentoring health facility nurses on PMTCT guidelines and use of PDSA cycles to improve PMTCT service delivery ➢ Providing and maintaining POC devices for CD4 testing in ANC ➢ Supporting EID and CD4 specimen courier services ➢ Supporting HIV test-kit and ARV stock management and redistribution

The CPM, involved cycles of planning and implementing activities designed to address national programme gaps identified in the previous year, collecting and analyzing quarterly CPI data in the new year, conducting quarterly programme reviews, planning and implementing recommended CP activities at different sites; conducting annual CPI data reviews at the end of the year and planning the next year’s activity plans [28, 29]. Programme reviews discussed district and facility level performances of each CPI and recommended new “course correction” CPAs to address identified gaps. The consortium identified course correction CPAs from published implementation research and quality improvement tested changes. Staff from MoHCC national PMTCT programme, DFPs and consortium staff participated in the programme review meetings. DFPs assisted health facilities to implement the course correction CPAs. During the annual review and planning process, CIFF coordinated with other donors of Zimbabwe’s national PMTCT programme, including the United States Agency for International Development (USAID), United Nations Children’s Fund (UNICEF) and WHO.

### Data collection and management

We collected PMI data for the pre-implementation period (2002–2010), from MoHCC’s published PMTCT annual reports and CPI data from MoHCC District Health Information System (DHIS2). MoHCC used the DHIS2 electronic database to collect various health indicators from the country’s health facilities. Nurses recorded the women and infants that received PMTCT services in patient registers daily and compiled monthly summary statistics which they submitted to MoHCC’s district health information officers, who entered the data into the DHIS2. We collected PMI and CPI data for the project implementation period (2011–2015) from EGPAF’s project database. DFPs collected PMI data using quarterly service availability assessment visits and CPI data from health facility DHIS2 reports on Microsoft Excel tools, and submitted the data to EGPAF-Zimbabwe’s country office where monitoring and evaluation officers verified and uploaded the data into the Foundation’s project database.

### Data analysis

**Fig. 2:**
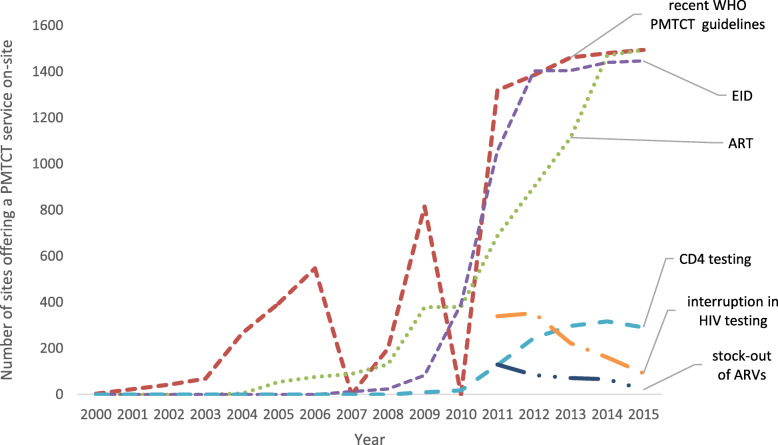
Number of sites offering or with interruptions in PMTCT services related to critical path indicators, 2000–2015

**Fig. 3:**
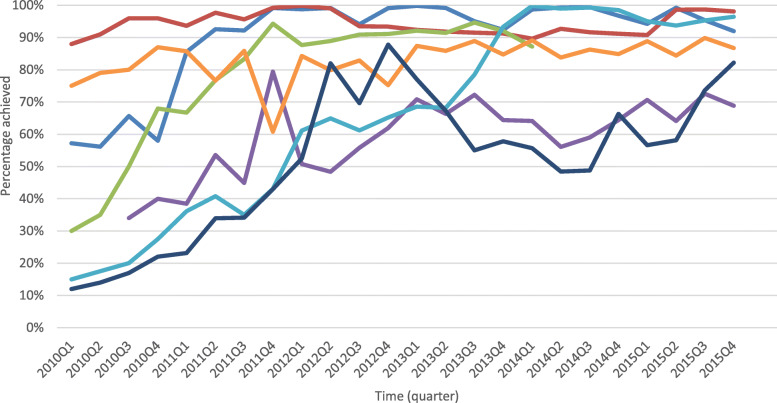
Trends in quarterly critical path indicator results, 2010–2015.  ANC bookings.  HIV testing.  AZT prophylaxis.  CD4 testing.  Mothers ART.  Infant NVP.  EID

We performed graphical trend analysis of the PMIs (see Fig. 2) and KPIs (see Fig. 3) data to assess changes in PMTCT service availability and uptake before and during the EGPAF-CIFF project. We calculated percentage change in the number of clients that received PMTCT services in the 5 years before and during the EGPAF-CIFF project and performed Pearson’s Chi2 test to compare the proportions that received PMTCT services in each CPI (Table 3). We used *p*-values < 0.05 to determine statistical significance of the differences.

**Table 3:** Comparison of number and percentage of women and infants receiving PMTCT services, before (2006–2010) and during (2011–2015) the EGPAF-CIFF CPM project

Indicator	Number of women and infants receiving PMTCT services (numerator) and percentage, out of those eligible (denominator) in pre-intervention and intervention period		% change in ***number*** of women & infants receiving PMTCT services before and during the project^a^	Person’s Chi2 test *p*-value comparing % women & infants receiving PMTCT services before and during the project
	Pre-Intervention Period (2006–2010)	Intervention Period (2011–2015)		
Pregnant women booked for ANC	59% (1,176,003/1,985,076)	97% (2,048,283/2,105,205)	74%	< 0.001
Pregnant women tested for HIV in ANC	80% (941,293/1,176,003)	99% (1,953,814/1,972,936)	108%	< 0.001
HIV-positive pregnant women dispensed ARVS	24% (34,330/141,064)	74% (123,547/167,839)	260%	< 0.001
HIV-positive pregnant women CD4 tested	11% (15,543/141,064)	62% (123,578/197,788)	695%	< 0.001
HIV-positive pregnant women initiated ART	2% (3287/141,064)	42% (83,494/197,788)	2440%	< 0.001
HIV-exposed infants initiated ARV prophylaxis	70% (98,898/141,064)	85% (244,814/287,543)	148%	< 0.001
Infants HIV tested using DNA PCR < 2 months	9% (12,494 /141,064)	58% (167,291/287,543)	1239%	< 0.001

^a^Measures the change in the numbers provided PMTCT services between the pre-intervention and intervention period

We performed the *sktest* on quarterly CPI uptake percentages from 2010 to 2015 to check if the data were normally distributed, as the statistical tests that we used required the data to follow a normal distribution [30]. We then performed the regression discontinuity (RD) analysis (Table 3), to test if the interventions implemented (Table 1) increased the CPIs. The sktest requires a minimum of eight observations for a variable and presents two normality tests based on skewness and kurtosis then combines the two into an overall normality test statistic [30]. Variables with *p*-values above a selected threshold (usually 0.05) are considered to be normally distributed [30]. We considered indicators (variables) with *p*-value above 0.05 to be normally distributed. We performed the RD analysis on each CPI based on the fact that the project implemented separate activities to improve each CPI. RD analysis splits the curve of an indicator which is plotted against time (see Fig. 3) into two segments, splitting the curve at the point in time where the intervention was introduced. The RD analysis then estimates each segment of the indicator’s curve with a trend line and computes the gradients (with their 95% confidence intervals (CIs)) of the two trend lines (i.e. the steepness of the trend lines for the two time periods – before and during the intervention). The 95% CIs are used to determine statistical significance of differences between gradients of the two lines [31, 32]. Overlapping CIs will mean that the CPI did not increase after introducing the intervention. We performed all statistical analysis using Stata version 15.0 [24, 33].

## Results

PMTCT service availability improved drastically during the EGPAF-CIFF critical path driven project (Fig. 2). Before the project, 2006 WHO PMTCT guidelines were rolled out to 815 sites from 2006 to 2010 (*n* = 1560), i.e. the 2006 guidelines were rolled to 52% of the sites in 4 years. However, 2010 guidelines (implemented through EGPAF-CIFF project) were rolled out to 1320 sites from January to September 2011, a period of 9 months. Similarly, 2013 WHO guidelines were rolled out to 1385 sites from January to May 2013, a period of 5 months. EID (collecting DBS from HIV-exposed infants for DNA PCR testing, receiving results and issuing to caregiver) was rolled out to 392 sites from 2006 to 2010 and from 392 to 1403 between 2011 and 2012. Interruptions in HIV testing (failing to offer HIV testing for at least 1 day because of absence of a trained tester or stocking out of HIV test-kits) declined from 38% (over 300 out of 815 sites) in March 2011 to 2% (30 out of 1495) in September 2015 (*p* < 0.001).

PMTCT service uptake improved considerably during the project (Fig. 3). The number of women and infants receiving PMTCT services increased significantly, comparing the pre-intervention and intervention period. The highest increase was recorded in infants tested for HIV using DNA PCR – 1239%, followed by women assessed for ART eligibility by CD4 count – 695% and eligible pregnant women initiated ART – 580% (by 2013 when “Option A” ended). The proportion (number who received the service out of those eligible) of women booked for ANC increased from 59 to 97% and proportion tested for HIV in ANC increased from 80% to about 99% (*p* < 0.001). The proportion of HIV-positive pregnant women receiving AZT prophylaxis increased from 24 to 74% by 2013 when “Option A” ended (*p* < 0.001) (Table 3).

All CPIs, except ART initiation of pregnant women in ANC (*p* = 0.0024), were normally distributed (*p* > 0.05). However, RD analysis applied to this indicator without violating the normality assumption because of the large sample size involved [34, 35]; hence, we performed RD analysis on all CPIs. The RD analysis showed that the interventions implemented on the different CPIs (Table 1) improved all the CPIs except for HIV testing in ANC and infant ARVs (Table 4).

**Table 4:** Regression discontinuity results for critical path indicators; 2010–2015

Indicator	***P-***value for Sktest of normality	Slope of linear curve before introducing intervention (β1)	Slope of linear curve after introducing intervention (β2)	Decision
	***P-***value*	Slope (95% CI)	Slope (95% CI)	
ANC Bookings	0.3434	0.001 (−0.005–0.006)	0.310 (0.220–0.401)	β_2_ > β_1_ > 0
HIV Testing	0.1542	−0.001 (− 0.004–0.002)	0.029 (− 0.023–0.812)	^a^
AZT Prophylaxis	0.8391	0.142 (− 0.002–0.030)	0.271 (0.108–0.443)	β_2_ > β_1_ > 0
CD4 Testing	0.0560	0.008 (0.001–0.015)	0.168 (0.040–0.296)	β_2_ > β_1_ > 0
Mothers’ ART	0.0024	0.005 (−0.003–0.014)	0.160 (0.036–0.282)	β_2_ > β_1_ > 0
Infant ARVs	0.3617	0.007 (0.002–0.012)	−0.064 (− 0.144–0.017)	^a^
EID	0.1502	0.010 (− 0.002–0.022)	0.309 (0.107–0.511)	β_2_ > β_1_ > 0

* Indicator meets normal distribution criteria if P > 0.05 or sample is large (> 3000 in all indicators, Table 3*)*

^a^ *95% CI of β*_*1*_ *and β*_*2*_ *overlapping or β*_*2*_ *< β*_*1*_ *hence no difference in slope between the two segments of the indicator’s curve, before and after the start of interventions*

## Discussion

Our study demonstrates that the CPM is one strategy that can be used to effectively scale up and optimise PMTCT programmes in sub-Saharan Africa where MTCT rates remain unacceptably high. In PMTCT, the efficacy of ARV regimens used determine the reduction of the MTCT rate of the country. However, the efficacy of ARV regimens depends on the extent of their roll-out and their uptake in the population. The CPM, in this case, helped to rapidly roll-out and to optimise the use of the new WHO ARV guidelines in the country.

The EGPAF-CIFF program used the CPM to roll-out 2010 and 2013 PMTCT guidelines to nearly all MNCH facilities of the country within one year. Only non-functional clinics did not implement the new guidelines. Previously, Zimbabwe took more than 4 years to introduce new WHO PMTCT guidelines and rolled them out to only 50% of the MNCH facilities in the country [10]. The rapid roll-out was achieved because activities implemented, such as training nurse HIV testers, supporting sites to order ARVs and redistributing ARVs from one facility to another, procuring and supplying CD4 testing devices, supporting EID courier services and other activities directly improved PMTCT service availability. Consequently, the number of women and children who received PMTCT services increased by two to fourteen-fold comparing the five-year pre- and intervention periods. Similarly, the uptake of all PMTCT services increased significantly during the intervention period.

Various studies corroborate our findings. D’Aquilla and Falconer noted that the CPM can be used to implement public health programmes in ways that optimises health outcomes [15, 18]. Similarly, Paintsil and Andiman, in a systematic review, observed that increased coverage of more efficacious ARV regimens is a key success factor of PMTCT programmes in resource-limited countries. The barriers to success of PMTCT programmes noted by the authors are lack of health care infrastructure, slow integration of PMTCT services into traditional MNCH services, limited manpower, limited funding, competing priorities against limited public health budgets, low ART and MTCT programme coverages, low coverage of PCR-based diagnosis for paediatric HIV and other factors [36]. In their systematic review, Ambia and Mandala found the use of community health workers, tracking HIV-positive women and training midwives in PMTCT integration into routine pregnancy and infant care and lab courier system for CD4 counts to increase PMTCT uptake [3]. Use of the CPM addressed the challenges identified in these reviews and implemented continuous course corrections that included the recommendations of these studies to the success of Zimbabwe’s PMTCT programme.

The RD analysis showed that the interventions implemented for ANC bookings, maternal AZT prophylaxis, CD4 testing, mothers’ ART initiation and EID significantly increased performance for these indicators. The interventions are similar to those recommended in Ambia and Mandal and barriers identified by Paintsil and Andiman in their systematic reviews [3, 36]. RD analysis does not compare results of different interventions but determines if the intervention introduced changed the related indicator results [31, 32]. Knowledge of the various projects implemented in the country at that time enables the attribution of results of the different indicators to the activities of this project. To the best of our knowledge, no other PMTCT support had comparable scale and intensity as the EGPAF-CIFF project.

RD analysis showed no significant increase in HIV testing of pregnant women and infant ARV prophylaxis uptake, likely for two reasons. Although the EGPAF-CIFF project expanded PMTCT services offered using new WHO guidelines from 50% to nearly 100% of MNCH facilities in Zimbabwe, HIV testing uptake in the 50% facilities was already high at about 90% [10]. Consequently, the CPM increased the percentage of sites offering PMTCT using new WHO guidelines but did not drastically increase the uptake of HIV testing in ANC, which was already high. RD analysis may have not detected the impact of interventions implemented on the infant ARV indicator because of challenges with the way that the indicator was measured. The numerator was the number of HIV-exposed infants dispensed ARV prophylaxis and the denominator was the number of HIV-positive pregnant women identified in ANC during each quarter. Given that infant ARV prophylaxis dispensing was done at delivery while the HIV-positive mothers would be identified at ANC booking, which occurred from 14 weeks’ gestation, the women counted in the denominator were not necessarily the mothers of the HIV-exposed infants started on ARV prophylaxis that quarter. This compromised the sensitivity of the indicator, making it difficult to detect the impact of the interventions implemented on it.

We believe that the CPM approach contributed to the rapid decline in Zimbabwe’s MTCT rate from 2011 to 2015. Zimbabwe’s MTCT rate declined from 22 to 6.4%, close to the UNAIDS target of 5% by 2015. Although UNAIDS 2015 spectrum estimates indicate that the MTCT rate was already declining from 31% in 2000/2002 to 22% in 2010, the MTCT rate declined more rapidly from 20% in 2011 to 6.4% in 2015 [20]. Similarly, population-based PMTCT impact studies of Zimbabwe found drastic declines in the MTCT from 10% in 2012 to 4.8% in 2014 [19].

The drastic declines observed by UNAIDS modeling estimates and PMTCT impact studies occurred during the EGPAF-CIFF project. The early declines observed in the MTCT rate may be associated with high HIV mortality, behavior change associated with HIV mortality and prevention efforts and the impact of ART from 2004 onwards, as noted by Mahomva et al. [37]. UNAIDS Spectrum modeling shows a stabilization of the MTCT rate at 6–7% from 2016 to 2019. This suggests that the end of the EGPAF-CIFF project, which scaled down substantially in 2015 and ended in September 2017, may have removed the strong driving force for continued decline of the MTCT rate. Continuation of PMTCT support at the same intensity as 2011–2015 may have reduced the MTCT rate further. The stabilization trend may also indicate the challenge of reaching the last mile of public health targets, which takes more resources for reduced impact.

The CPM is one of various QI approaches that can be used to rapidly achieve programme targets. In other literature, the CPM is also referred to as the Program Evaluation and Review Technique (PERT) [15, 17]. Other approaches include the rapid results initiative (RRI) or rapid results approach (RRA), results-based management (RBM), results-based financing (RBF), continuous quality improvement (CQI), and PDSA cycles. Governments largely use RRI, RRA and RBM strategies to accelerate economic recovery and development programmes [38–41]. Many sub-Saharan Africa countries use the three strategies in various government programmes [38–41]. Challenges faced with these approaches include poor management control systems in the public sector, difficulties to change public sector culture, political influence in public sector management and resource inadequacies [40].

To improve health indicators, governments and development partners mostly use RBF, CQI and PDSA cycles [38, 41, 42]. These approaches are more adapted to health systems. Among the three approaches commonly used in health programmes, the CPM has the comparative advantage that it incorporates elements of the other three. The budgeting and financing approach in the CPM incorporates the RBF concept of performance-based resource allocation, while the activity implementation incorporates CQI and PDSAs [15, 17]. The inclusion of PDSA cycles in the CPM encourage programme implementers to use their data and drive their own facilities’ performance. The CPM makes programme performance guide resource allocation in activity budgets.

The CPM provides a systematic method of engagement with government and key health development partners, through joint annual planning and review meetings. In Zimbabwe, the government adopted various new policies recommended during the CPM, including incorporating PMTCT in VHW training curriculum, introducing nurse-led ART initiation, introducing clinical mentorship and ART initiation in MNCH clinics and adopting QI strategies in all HIV programmes using PSDA cycles. Lyons and Pillay documented the impact of approaches used in the EGPAF-CIFF project on country and community leadership to achieve the global plan for elimination of mother-to-child transmission of HIV target [43]. The CPM facilitated this country, community and facility leadership engagement during the project.

This paper has described the use of the CPM in a public health programme where application and documentation of the method is limited. Following the near achievement of the UNAIDS MTCT rate target of 5%, Zimbabwe is applying for WHO ‘path to elimination (PTE)’ award. The WHO PTE award is for countries that have made significant progress towards achievement of the elimination target [44]. CIFF leveraged the lessons of the CPM in Zimbabwe’s Hurungwe district, in the “Accelerating Children’s HIV/AIDS Treatment (ACT) initiative” in 2015 [45]. Together with the President’s Emergency Plan for AIDS Relief (PEPFAR), CIFF also leveraged the CPM in the initiative to improve HIV testing, treatment and care for children and adolescents in 9 priority countries – namely, Cameroon, Democratic Republic of Congo, Kenya, Lesotho, Malawi, Mozambique, Tanzania, Zambia, and Zimbabwe from 2016 to 2019 [46].

The limitations of this study are that it uses retrospective programme data. The authors had limited control on the quality of the data analysed. The EGPAF-CIFF project coincided with the introduction of more efficacious PMTCT regimens like Option B+. This makes it difficult to attribute the decline of Zimbabwe’s MTCT rate entirely to use of the CPM. However, the CPM played a clear role to rapidly roll-out and optimize uptake of the new regimens, which was a challenge in the roll-out of previous regimens. Without this rapid roll-out and the optimization of uptake, it would have been difficult for the regimens to have an impact on the MTCT rate. The strength of this paper is that it is based on field-based public health experience of implementing the CPM and uses a wide range of data to demonstrate the benefits of employing the approach. The approach has been leveraged also in follow-up CIFF projects implemented in other countries, which is evidence of its growing successes.

## Conclusions

The CPM is an innovative strategy that helps to rapidly scale-up, rationalize resource use and optimise public health programme outcomes. It is able to help operationalize global public health guidelines. Through its approach to resource allocation, which targets the less performing areas of the programme, the CPM is a particularly useful and effective tool in low resource settings. These settings are often faced with acute limitations to rapidly implement public health strategies and activities. We recommend global and national health programmes to use the CPM, among other strategies, to rapidly achieve public health goals.

## Data Availability

The data used in this manuscript are available in MoHCC’s DHIS2 database, PMTCT annual reports from 2009 to 2015 and MoHCC national HIV estimates reports. The datasets used in the manuscript can be available from the corresponding author on a reasonable request. The MoHCC DHIS2 database is accessible by permission from the Ministry. Some annual reports and national HIV estimates reports are available online (https://zdhr.uz.ac.zw/xmlui/handle/123456789/1423; https://www.pedaids.org/wp-content/uploads/2017/11/ZIM2014AR_Aug5.pdf; https://info.undp.org/docs/pdc/Documents/ZWE/MOHCC%20GF%20Quarterly%209%20Final%20Report%2020%2004%2016%20Jan%20to%20March%202016.doc; https://b.3cdn.net/glaser/d0b2becd60d22576e0_pjm6boguj.pdf; https://www.unaids.org/sites/default/files/country/documents/ZWE_narrative_report_2016.pdf;http://nac.org.zw/wp-content/uploads/2019/01/Zimbabwe-HIV-Estimates-Report-2018.pdf).

## Abbreviations

AIDS: Acquired immuno-deficiency syndrome
ANC: Antenatal care
ARV: Antiretroviral
ART: Antiretroviral therapy
AZT: Zidovudine
CD4: Cluster of differentiation 4
CI: Confidence interval
CP: Critical path
CPA: Critical path activity
CPI: Critical path indicator
CPM: Critical path method
CIFF: Children’s Investment Fund Foundation
CQI: Continuous quality improvement
DBS: Dried blood spot
DFP: District focal person
DHIS2: District health information system version 2
DNA: Deoxyribonucleic acid
DNO: District Nursing Officer
EGPAF: Elizabeth Glazer Paediatric AIDS Foundation
EID: Early infant diagnosis (of HIV)
HIV: Human immune-deficiency virus
MTCT: Mother-to-child transmission (of HIV)
MoHCC: Ministry of Health and Child Care
MNCH: Maternal, neonatal and child health
MRCZ: Medical Research Council of Zimbabwe
NVP: Nevirapine
PEPFAR: President’s Emergency Plan for AIDS Relief
PDSA: Plan-do-study-analyse
PMI: Programme management indicator
PMTCT: Prevention of mother-to-child transmission (of HIV)
POC: Point-of-care
PCR: Polymerised chain reaction
PTE: Path to elimination
QI: Quality improvement
RBF: Results-based financing
RRA: Rapid results approach
RRI: Rapid results initiative
RBM: Results-based management
RD: Regression discontinuity
Sd: Single dose
Sd NVP: Single dose Nevirapine
UNAIDS: United Nations Joint Program of HIV/AIDS
UNICEF: United Nations Children’s Education Fund
USAID: United States Agency for International Development
VHW: Village Health Worker
WHO: World Health Organization
